# Supplementary material for: Propagation-adaptive 4K computer-generated holography using physics-constrained spatial and Fourier neural operator
Source: Nat Commun. 2025 Aug 20;16:7761. doi: 10.1038/s41467-025-62997-z (PMC12368012; doi:10.1038/s41467-025-62997-z)
Supplement: Supplementary file 1 — Supplementary Information [file 41467_2025_62997_MOESM1_ESM.pdf]

Supplementary of

Propagation-adaptive 4K computer-generated holography using physics-constrained spatial and Fourier neural operator

Ninghe Liu,[1] Kexuan Liu,[2] Yixin Yang,[2] Yifan Peng,[3] and Liangcai Cao[1,2,]*

[1]Weiyang College, Tsinghua University, Beijing 100084, China.

[2]Department of Precision Instrument, Tsinghua University, Beijing 100084, China.

[3]Department of Electrical and Electronic Engineering, The University of Hong Kong, Hong Kong SAR, China.

Email: clc@tsinghua.edu.cn

**Supplementary Note 1**	Information flow and receptive field analysis
**Supplementary Note 2**	Justification for circular constraint through model parameter counts
**Supplementary Note 3**	Fourier embedding and sampling analysis
**Supplementary Note 4**	Illustrations of training and testing dataset arrangement
**Supplementary Note 5**	Comparison between SFO-solver and non-learning CGH algorithms
**Supplementary Note 6**	Depth extension test and ablation study of Fourier embedding
**Supplementary Note 7**	Additional results for resolution chart holographic projection
**Supplementary Note 8**	Phase extraction methods for multi-plane 3D display
**Supplementary Note 9**	Additional results on more complex 3D scene holographic display
**Supplementary Note 10**	Discussions on the PO-CGH synthesized by SFO-solver

Supplementary Note 1: Information flow and receptive field analysis

In this note we analyze the optical information flow in different configurations that may happen in our experiments and demonstrates SFO-solver's efficient RF matching backbone compared to other networks. Our experimental prototype includes the phase-only SLM (HOLOEYE GAEA-2) with the pixel pitch of 3.74 μm and the resolution of 2160×3840. The propagation distance is set from 85 mm to 115 mm, and the illumination wavelengths are 638 nm, 520 nm and 450 nm for RGB laser, respectively. Assuming that the SLM pixel can be seen as a square plane with uniform phase, the PSF needs to be treated in a pixelized manner, as schematically shown in Fig. S1 (green illumination as an example).

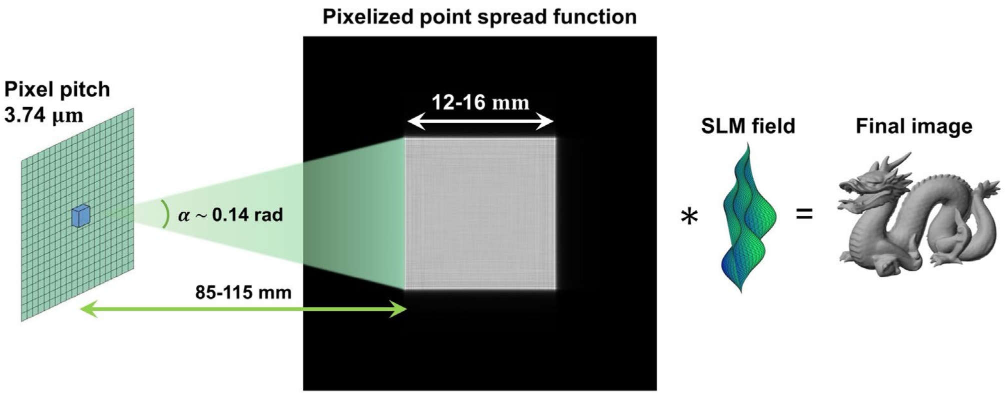

Fig. S1 | Schematics of pixelized PSF analysis and the image formation in the diffraction process. Images reproduced from the Stanford 3D Scanning Repository[1] (https://graphics.stanford.edu/data/3Dscanrep/).

The diffraction angle of a single pixel, the PSF spatial width and the required RF to address the information flow matching is estimated in Table S1. Since the input intensity image has the pixel size of 2160×3840, the requirement of digital information flow essentially indicates a global RF for the neural solver.

Table S1. Optical information flow and the corresponding digital receptive field

Configuration	Optical information flow		Digital information flow
	Diffraction angle	PSF width	Required receptive field
R / 85-115 mm	0.17 rad	14.5-19.6 mm	3877-5240
G / 85-115 mm	0.14 rad	11.8-16.0 mm	3155-4278
B / 85-115 mm	0.12 rad	10.2-13.8 mm	2727-3690

Notably, other network structures such as self-attention[2] have also been utilized to address this RF matching problem in CGH optimization. However, despite the self-attention layer is able to achieve great RF expansion compared to traditional CNN layer, its computational memory requirements still grow quadratically as the image resolution increases. This makes it unfeasible to implement full self-attention for 4K image inputs. In contrast, the Fourier operator requires computational memory that scales linearly with image resolution. Moreover, when a circular constraint is applied, this scaling can be further reduced to a square-root level.

Supplementary Note 2: Justification for circular constraint through model parameter counts

In this note we provide a justification for the necessity of the circular constraint applied in SFO-solver by analyzing the associated model parameter counts. Specifically, SFO-solver employs a 3-layer MLP to perform the mapping from the embedded distance input to the circular Fourier multiplier, as illustrated in Fig. S2 (a). Based on the appropriate sampling rate that we calculate in Section 4.1 of the main manuscript ($N_{sample} \sim 500$), we estimate that the circular constraint leads to a 10^5-fold reduction in parameter counts and model size. Without this constraint, implementing the distance encoder would require an estimated 20 TB of GPU memory, whereas the circular constraint reduces this requirement to just 80 MB—well within the capacity of most commercially available GPUs.

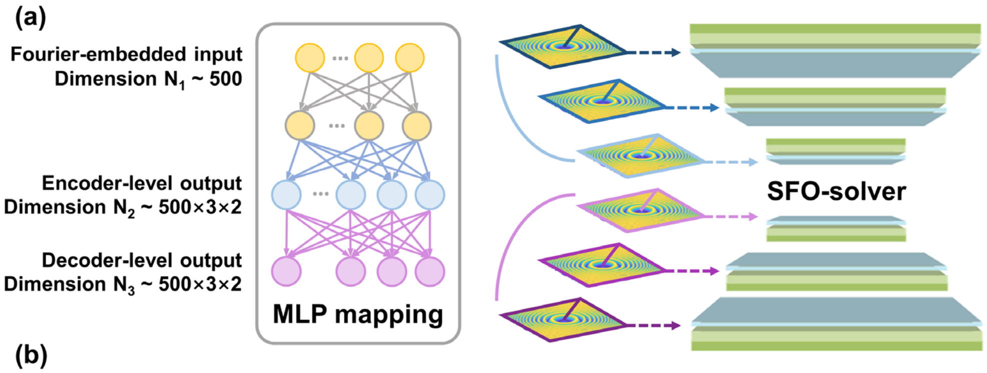

MLP learning	Output dimension	MLP parameter counts	Estimated size
w circular constraint	~ $500 \times 3 \times 2$	~ 2×10^7	~ 80MB
w/o circular constraint	~ $500^2 \times 3 \times 2$	~ 5×10^{12}	~ 20TB

Fig. S2 | (a) MLP structure for distance encoding. (b) Parameter counts with or without circular constraint.

Supplementary Note 3: Fourier embedding and sampling analysis

In this note we provide a theoretical guidance of the parameters chosen in SFO-solver's Fourier embedding operation. As illustrated in the main manuscript, for a typical MLP model to efficiently map the input coordinate to high-frequency features of output filter $\mathrm{H}(z)$, a frequency-matching modulation $\zeta(z)$ is conducted before MLP processing. Different from the positional encoding in neural rendering[3,4], where the modulation frequency starts from zero, the frequency range in our case is selected to match with that of $\mathrm{H}(z)$, as shown in Fig. S3(a).

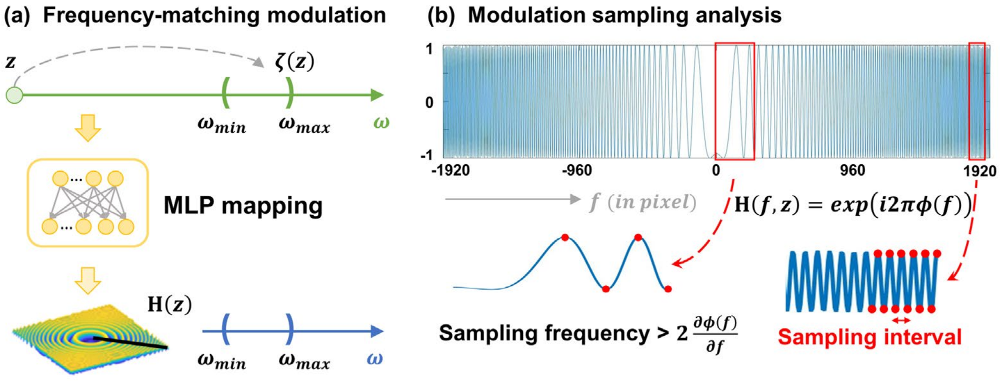

Fig. S3 | (a) Frequency modulation with respect to distance z and (b) Sampling analysis for Fourier embedding operation.

In the Fourier branch of the SFO-solver, $\mathrm{H}(z)$ is treated as a discrete multiplier. In order to determine how many neural nodes are actually required to represent this multiplier, we conduct the sampling analysis illustrated in Fig. S3(b). Because SFO-solver is designed to allow a free distance range of 30 mm, $\mathrm{H}(z)$ only needs to address the influence of this 30 mm propagation into our neural solver. The rest information is incorporated in the global multiplier H in Fig. 2 (c) of the main manuscript. This treatment follows the concept of midpoint hologram in "Tensor holography[5]", where their CNN model only needs to address the 6 mm optical path of the 3D volume. For the AS filter $\mathrm{H}(f, z)$ with $z \in [-15, 15]$ mm, its maximum local frequency determines the minimum sampling frequency.

$$\mathrm{H}(f,z) = e^{i2\pi\phi(f)} = e^{i2\pi\,\Delta z\sqrt{\frac{1}{\lambda^2}-f^2}} \tag{S1}$$

$$\vartheta_{sample} > 2\frac{\partial\phi(f)}{\partial f} = 2\frac{\Delta z\,\lambda f}{\sqrt{1-\lambda^2 f^2}} \tag{S2}$$

Assuming uniform sampling and bilinear extrapolation of $\mathrm{H}(f,z)$, and substituting $f_{max} = \sqrt{2}/\Delta x$ into Eq. (S2), the minimum sampling number should be

$$N_{sample} = f_{max} \times \vartheta_{sample} = 4\frac{\Delta z\,\lambda}{\Delta x\,\sqrt{\Delta x^2-2\lambda^2}} \tag{S3}$$

In our configuration, where SLM pixel pitch size $\Delta x = 3.74$ µm and Δz =15 mm, the N_{sample} for red (638 nm), green (520 nm) and blue (450 nm) channels are calculated to be 689, 560 and 484, respectively. It's also important to note that the AS filer in Eq. (S1) exhibits a frequency variation on the order of $O(f^2)$, implying that, under the same sampling conditions, SFO-solver can achieve a fourfold increase—across a 120 mm distance range—in precise focus control for a 1080p commercial SLM (typically 8µm pixel pitch).

$$e^{i2\pi\,\Delta z\sqrt{\frac{1}{\lambda^2}-f^2}} \approx e^{i2\pi\frac{\Delta z}{\lambda}}\,e^{-i\pi\,\Delta z\lambda^2 f^2} \sim e^{i\,[\Delta z\,O(f^2)]} \tag{S4}$$

The quadratic relation can also be seen from Eq. (S3), where reducing the pixel size Δx by half would lead to a fourfold increase in the sampling number N_{sample}.

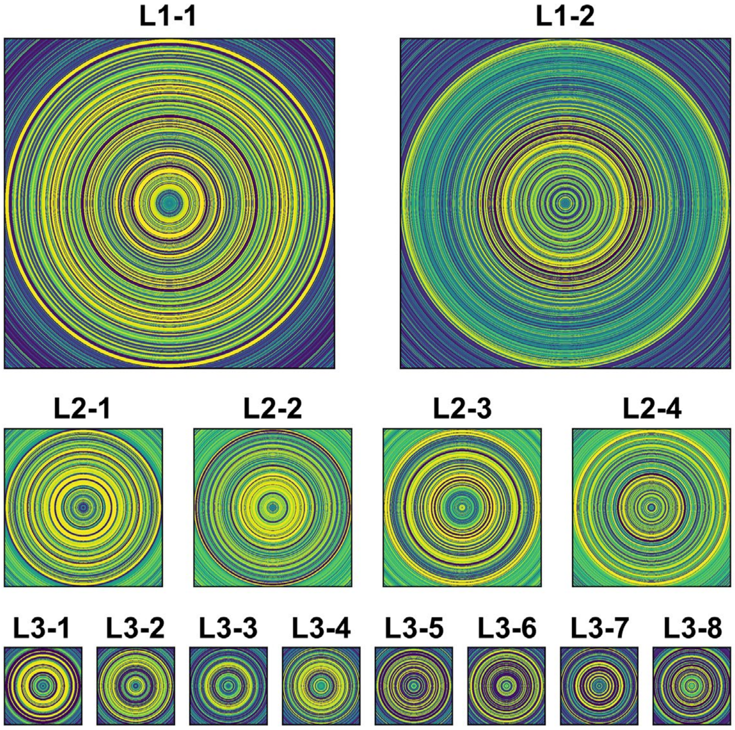

Fig. S4 | Full example visualizations of the learned circular multipliers in SFO-solver's encoder-levels. Input distance is 85mm in this case. The expression of 'L*x*-*y*' indicates the Fourier multiplier at SFO-solver's hierarchical level *x* and dimension *y*.

Figure 2(d) in the main manuscript visualizes some of the learned circular multipliers in SFO-solver's intermediate layers. Here we provide full visualizations of the encoder-level multipliers when input distance is 85mm. We argue that this high-frequency structure validates the use of Fourier embedding given that MLP has been proved to struggle in learning high-frequency functions in many impactful researches[3,4]. Additionally, in Note 6 we'll provide the ablation studies regarding Fourier embedding and conclude that such Fourier feature is actually essential for SFO-solver to learn actual physics.

Supplementary Note 4: Illustrations of training and testing dataset arrangement

In this note we provide detailed illustrations of training and testing data for SFO-solver. As explained in the main manuscript, training images are from DIV2K trainset (image number: 0001-0800) and resized to the target resolution before fed into the network. For each input image, ten distances are randomly sampled between 85 mm and 115 mm to form the input 8,000 data pairs. Because the natural images in DIV2K dataset includes all frequency components that affect the forward optical problem, the inverse solver should generalize well. As shown in Fig. S5, our test data consists of multi-style images, including animation scenes (from Big Buck Bunny video), DIV2K testset (image number after 0800), and some structured emblems. In Ref. [12], we have uploaded the test images as well as 3-channel test results for readers to play with.

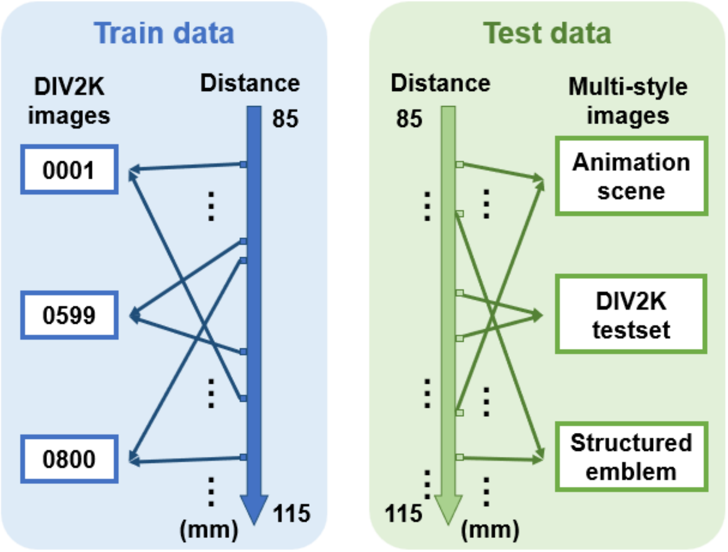

Fig. S5 | Illustrations of the training and testing data. Training images are from resized DIV2K trainset and test data are composed of multi-style images.

Supplementary Note 5: Comparison between SFO-solver and non-learning CGH algorithms

In addition to the previous learning-based CGH algorithms, we also compare SFO-solver with other non-learning CGH algorithms, including DPAC[6] method and iterative optimization approaches such as GS[7] and SGD[8] algorithms, as shown in Supplementary Fig. S6. As we already demonstrate SFO-solver's distance consistency, here we only choose the distance 100 mm for algorithm comparisons. Among the four CGH algorithms, the best image quality can be achieved using SFO-solver (39.25 dB) with an average processing speed of 0.157 seconds per frame. In contrast, GS method (the blue line) is prone to get into stagnation when it encounters some local minima during optimization. While SGD (the green line) is able to match SFO solver in terms of image PSNR, it requires about 100 seconds of iterations to generate a single frame. DPAC seems to achieve an acceptable balance between algorithm runtime and performance in simulation, however, experimentally its performance is usually much worse because of the low optical efficiency, phase wrapping issue and filtering misalignment, as suggested in the Supplementary S6 of Ref. [10]. All algorithms are run on GPU and the running code is also modified from Ref. [10] and is uploaded in Ref. [12].

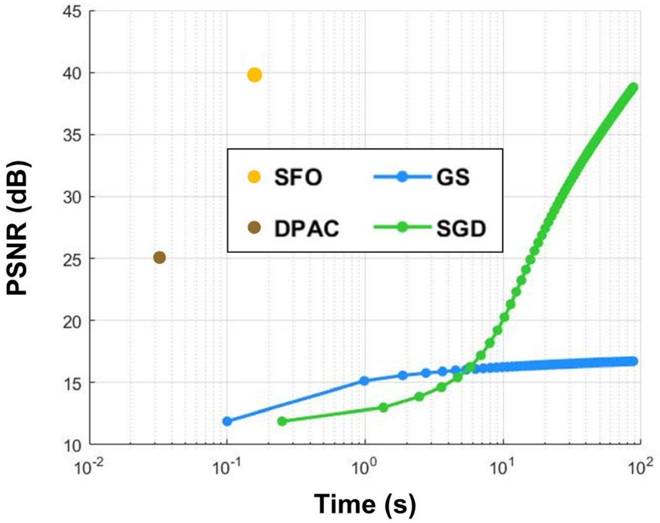

Fig. S6 | Comparison between SFO-solver and other non-learning CGH algorithms.

Supplementary Note 6: Depth extension test and ablation study of Fourier embedding

To evaluate the generalization ability of SFO-solver beyond its training range and to assess the role of Fourier embedding in distance encoding, we conducted a depth extension test using the same training and testing dataset. In this experiment, SFO-solver was trained on input distances between 85 mm and 115 mm, with and without Fourier embedding. During testing, we evaluated reconstructions of both networks across an extended range from 75 mm to 125 mm. As shown in Supplementary Fig. S7, the model equipped with Fourier embedding maintains a high average PSNR close to 40dB and SSIM above 0.98 within the training range and exhibits graceful degradation outside of it: even between 80 mm and 120mm, the PSNR remains above 30 dB, indicating acceptable reconstruction quality.

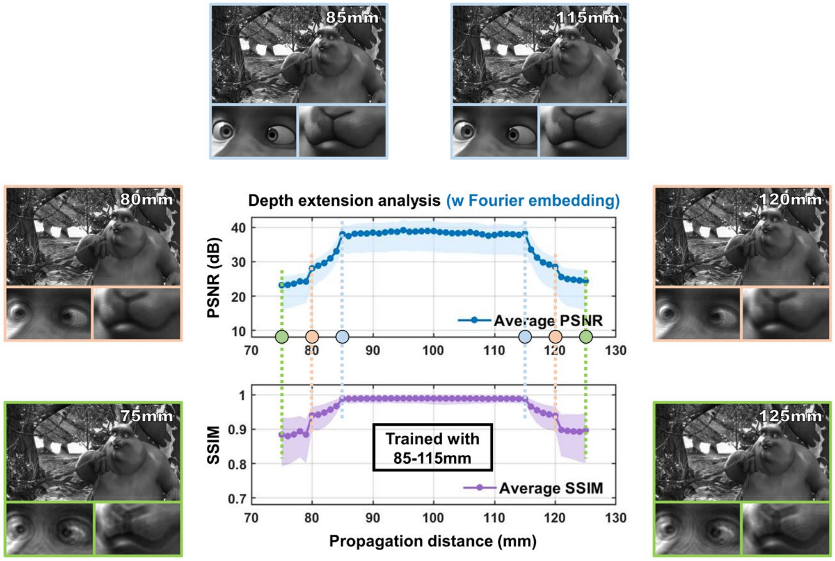

Fig. S7 | Depth extension analysis with Fourier embedding (green channel results). Images reproduced from www.bigbuckbunny.org (© 2008, Blender Foundation) under a Creative Commons licence (https://creativecommons.org/licenses/by/3.0/).

In contrast, the model without Fourier embedding experiences an abrupt quality drop at the training boundaries, with PSNR plummeting immediately to ~ 22dB at 84 mm and 116 mm. Supplementary Fig. S8 shows quantitative image quality evaluations across the full 50mm depth range. Supplementary Fig. S9 provides close-up comparisons near training boundaries (81–85 mm and 115–119 mm). SFO-solver with Fourier embedding captures subtle depth-

dependent variations and yields consistent image focus degradation, whereas the model without embedding exhibits unstable and incoherent reconstructions. These results confirm that Fourier embedding not only enables robust generalization, but also ensures that SFO-solver effectively learns the underlying physics of optical wave propagation.

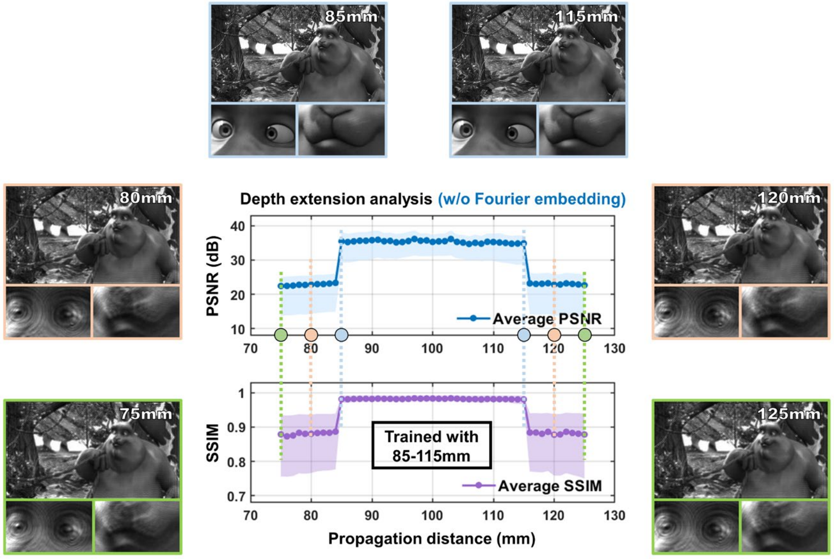

Fig. S8 | Depth extension analysis without Fourier embedding (green channel results). Images reproduced from www.bigbuckbunny.org (© 2008, Blender Foundation) under a Creative Commons licence (https://creativecommons.org/licenses/by/3.0/).

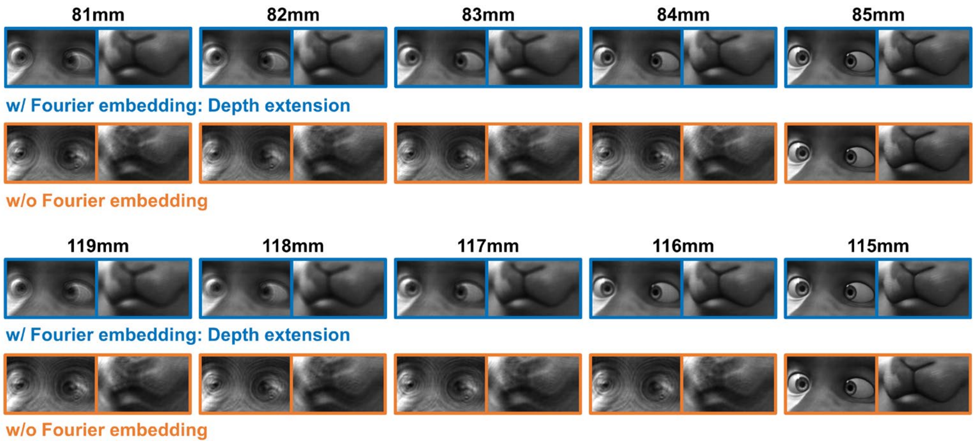

Fig. S9 | Close-up comparison of depth extension in the ablation study. Images reproduced from www.bigbuckbunny.org (© 2008, Blender Foundation) under a Creative Commons licence (https://creativecommons.org/licenses/by/3.0/).

Supplementary Note 7: Additional results for resolution chart holographic projection

Additional results for the resolution chart holographic projection at different wavelengths are listed in Fig. S10. In the optical experiments we observe a lower resolution in green and blue holographic projections, with approximately 34 μm peak-to-valley linewidth compared to 26 μm in red projections. However, the numerical results demonstrate excellent reconstructions with much finer details. This resolution discrepancy is attributed to optical misalignments and aberrations. The major experimental factors that would affect the optical resolution include: (1) Unwanted band clip in frequency filtering: When we filter out the zero-th order beam, some desired frequencies could be accidentally blocked by the slit; (2) Chromatic aberration of the 4f lens and slight misalignments.

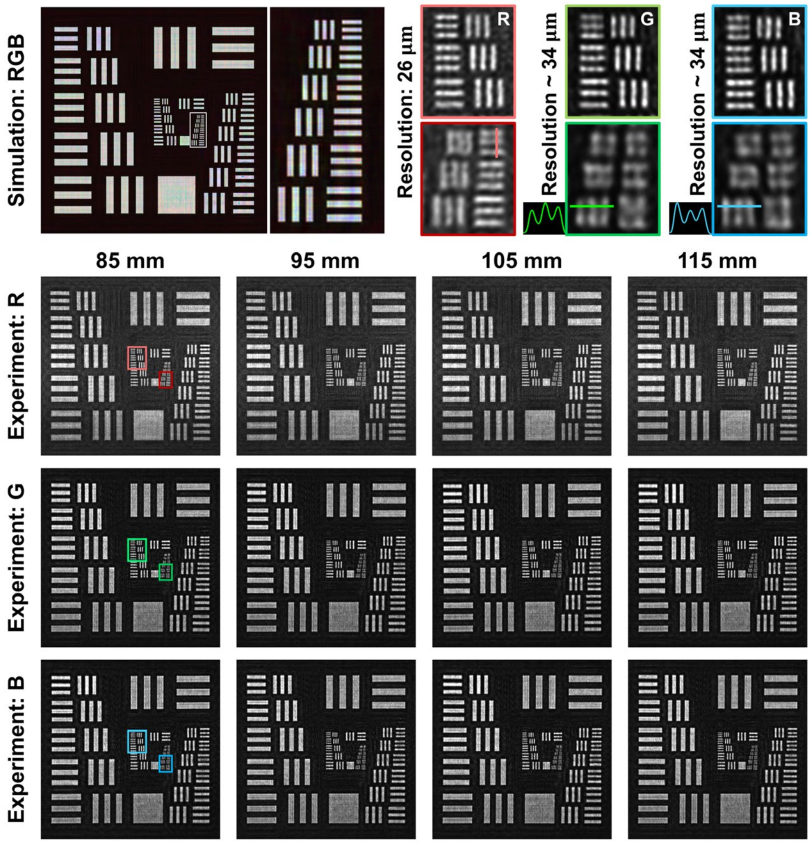

Fig. S10 | Holographic projection of the resolution chart at different wavelengths (all in-focus).

Supplementary Note 8: Phase extraction methods for multi-plane 3D display

SFO-solver enjoys a powerful capability of generating CGHs with flexible depth control. A promising application of this is to achieve adjustable depth control of multiplane holographic display. However, since the model is trained to synthesize 2D PO-CGHs corresponding to single propagation distances, a dedicated phase extraction strategy is required to merge multiple PO-CGHs into a single hologram for 3D display.

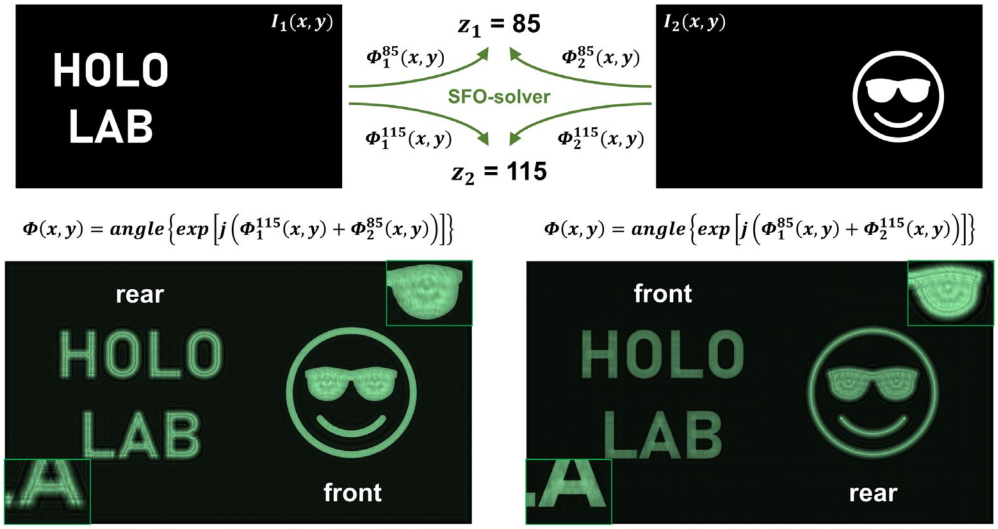

Fig. S11 | SFO-solver enabled multiplane 3D holographic display using layer-oriented phase extraction method.

Here we adopt a rather simple layer-oriented phase extraction method[9], as illustrated in Fig. S11. The input intensity images are first segmented and paired with their respective propagation distances. These intensity–distance pairs are then processed by SFO-solver in parallel batches. Since the dominant contribution to optical reconstruction arises from the phase profile, the resulting complex-valued fields from each depth are combined, and the final PO-CGH is obtained by extracting the global phase from the superposed complex field. We note that this straightforward method does not provide full 3D cues compared with more advanced focal stacks method. While we successfully demonstrate holographic display of more complex

natural scenes with depth occlusion in the following Note 9, future integration with advanced 3D graphics techniques may further enhance SFO-solver's applicability to intricate volumetric displays.

Supplementary Note 9: Additional results on more complex 3D scene holographic display

To further validate the effectiveness of SFO-solver in handling complex multi-plane holographic content, we present additional experimental results demonstrating the holographic display of natural 3D scenes with depth overlap, as shown in Fig. S12. Additional holographic projection results of natural scene pictures such as animals, vehicles and insects are uploaded in Ref. [12]. In these experiments, PO-CGHs are generated using SFO-solver with different input propagation distances while keeping the camera fixed at 85 mm. For each CGH, the resulting projection naturally emphasizes the target depth plane, as evidenced by the sharp reconstruction of in-focus objects and the blurred appearance of elements located on other planes. Insets with red and blue boxes highlight focused and defocused regions, respectively. These results confirm that SFO-solver can extend to more challenging 3D layouts, which highlight its potential for real-world volumetric display applications, even in scenarios that require accurate focus cues and layered spatial composition.

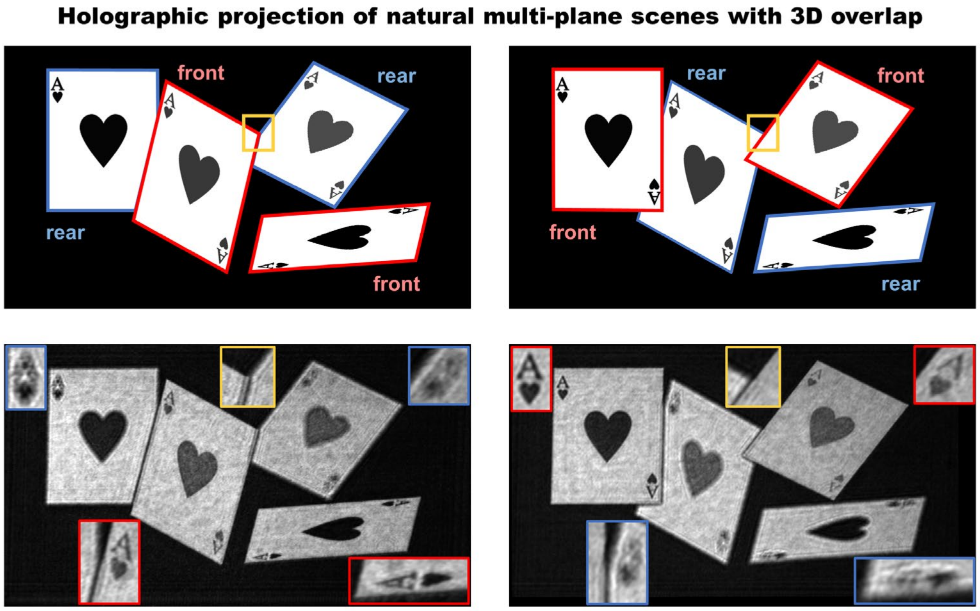

Fig. S12 | Holographic projection of natural multi-plane scenes with 3D overlap. Camera is fixed at 85mm and all focus/defocus cues are controlled by SFO-solver.

Supplementary Note 10: Discussions on the PO-CGH synthesized by SFO-solver

The PO-CGHs generated by SFO-solver exhibit certain detailed structures as shown in Fig. S13. This phase distribution is neither random nor smooth as in conventional CGH methods like GS and DPAC. In fact this feature has been discovered before by Peng, et.al in their supplementary material of "Neural holography[10]". And our experiments have justified that this indeed help reduce the reconstruction speckle.

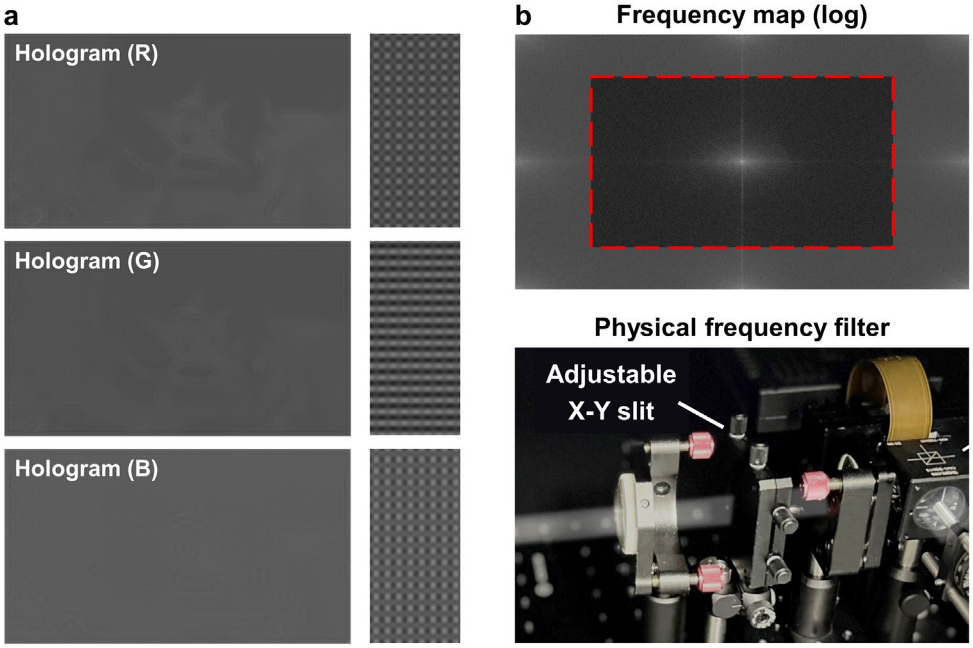

Fig. S13 | (a) Detailed structure of SFO-solver generated PO-CGHs. (b) Frequency filtering.

Another important experimental detail of our work is the frequency filtering, as shown in Fig. S13 (b). Because there is a band constraint (bounded by red box) manually added during network training, the frequency outside the constraint should be blocked in the experiment. In our setup, an adjustable X-Y slit is place at the central focal plane of the 4f system and act as a physical frequency selector. The advantages of this constraint has been explained using phase space analysis[11]. Experimentally we discovered it would speed up training convergence and reduce the reconstruction speckle.

Reference

1. Curless, B. & Levoy, M. in *Proceedings of the 23rd annual conference on Computer graphics and interactive techniques* 303–312 (Association for Computing Machinery, 1996).
2. Wei, Y. *et al.* Speckle-free holography with a diffraction-aware global perceptual model. *Photon. Res.* **12**, 2418-2423 (2024).
3. Mildenhall, B. *et al.* NeRF: representing scenes as neural radiance fields for view synthesis. *Commun. ACM* **65**, 99–106 (2021).
4. Tancik, M. *et al.* Fourier features let networks learn high frequency functions in low dimensional domains. *Advances in neural information processing systems* **33**, 7537-7547 (2020).
5. Shi, L., Li, B., Kim, C., Kellnhofer, P. & Matusik, W. Towards real-time photorealistic 3D holography with deep neural networks. *Nature* **591**, 234-239 (2021).
6. Maimone, A., Georgiou, A. & Kollin, J. S. Holographic near-eye displays for virtual and augmented reality. *ACM Trans. Graph.* **36**, Article 85 (2017).
7. Gerchberg, R. W. A practical algorithm for the determination of phase from image and diffraction plane pictures. *Optik* **35**, 237-246 (1972).
8. Peng, Y., Choi, S., Padmanaban, N., Kim, J. & Wetzstein, G. Neural Holography. In *ACM SIGGRAPH 2020 Emerging Technologies.* Article 8 (Association for Computing Machinery).
9. Zhao, Y., Cao, L., Zhang, H., Kong, D. & Jin, G. Accurate calculation of computer-generated holograms using angular-spectrum layer-oriented method. *Opt. Express* **23**, 25440-25449 (2015).
10. Peng, Y., Choi, S., Padmanaban, N. & Wetzstein, G. Neural holography with camera-in-the-loop training. *ACM Trans. Graph.* **39**, Article 185 (2020).
11. Chen, L., Tian, S., Zhang, H., Cao, L. & Jin, G. Phase hologram optimization with bandwidth constraint strategy for speckle-free optical reconstruction. *Opt. Express* **29**, 11645-11663 (2021).
12. Liu, N., Liu, K., Yang, Y., Peng, Y. & Cao, L. Propagation-adaptive 4K computer-generated holography using physics-constrained spatial and Fourier neural operator, CGH-SFO-solver, DOI: 10.5281/zenodo.15898299 (2025)
