# Supplementary material for: Propagation-adaptive 4K computer-generated holography using physics-constrained spatial and Fourier neural operator
Source: Nat Commun. 2025 Aug 20;16:7761. doi: 10.1038/s41467-025-62997-z (PMC12368012; doi:10.1038/s41467-025-62997-z)
Supplement: Supplementary file 2 — Description of Additional Supplementary Files [file 41467_2025_62997_MOESM2_ESM.pdf]

Description of Additional Supplementary Files

Supplementary Movie S1 | Hologram generation and simulated reconstruction of USAF target using the SFO-solver. The movie shows the generated hologram (top) and its corresponding simulated reconstruction (bottom, red channel) as the propagation distance varies from 85 mm to 115 mm. The hologram exhibits ripple-like variations as the input distance increases, while the close-up view of the reconstruction (inset) demonstrates fine details.

Supplementary Movie S2 | Hologram generation and simulated reconstruction of an animation scene using the SFO-solver. The movie shows the generated hologram (top) and its corresponding simulated reconstruction (bottom, green channel) as the propagation distance varies from 85 mm to 115 mm. The hologram exhibits ripple-like variations as the input distance increases, while the close-up view of the reconstruction (inset) demonstrates fine details.

Supplementary Movie S3 | Holographic refocusing at 115mm distance enabled by SFO-solver. As the camera shifts away from the original focal plane, the reconstructed projection of the Tsinghua emblem becomes progressively blurred. By feeding the updated defocus distance back to the SFO-solver, a new phase-only computer-generated hologram (PO-CGH) is rapidly synthesized and uploaded to the spatial light modulator (SLM), enabling real-time refocusing of the projected pattern.

Supplementary Movie S4 | Dynamic holographic refocusing enabled by SFO-solver. This movie demonstrates the dynamic refocusing process over a 30 mm range of camera displacement. As the camera shifts, causing the projection to become slightly defocused, the SFO-solver automatically engages to compensate for the distance change by recalculating and updating the hologram (in increments of approximately 5 mm). This process ensures the projection remains largely in focus throughout the entire range of movement.

Supplementary Movie S5 | Dynamic depth manipulation in a multi-plane holographic display using SFO-solver. This movie demonstrates the dynamic manipulation of relative object depth in a 3D scene while the camera remains fixed at 85 mm. Leveraging the phase extraction merging technique of two objects in a single frame, SFO-solver enables their spatial depth positions to be adjusted independently. This demonstrates the method's capability for creating interactive, multi-plane 3D displays without physically moving components.
